# Hole dynamics in a photovoltaic donor-acceptor couple revealed by simulated time-resolved X-ray absorption spectroscopy

**DOI:** 10.1063/1.5097653

**Published:** 2019-07-23

**Authors:** Khadijeh Khalili, Ludger Inhester, Caroline Arnold, Ralph Welsch, Jens Wenzel Andreasen, Robin Santra

**Affiliations:** 1 Department of Energy Conversion and Storage, Technical University of Denmark, Frederiksborgvej 399, 4000 Roskilde, Denmark; 2 Center for Free-Electron Laser Science, DESY, Notkestrasse 85, 22607 Hamburg, Germany; 3 The Hamburg Centre for Ultrafast Imaging, Luruper Chaussee 149, 22761 Hamburg, Germany; 4 Department of Physics, Universität Hamburg, Jungiusstrasse 9, 20355 Hamburg, Germany

## Abstract

Theoretical and experimental methodologies that can characterize electronic and nuclear dynamics, and the coupling between the two, are needed to understand photoinduced charge transfer in molecular building blocks used in organic photovoltaics. Ongoing developments in ultrafast pump-probe techniques such as time-resolved X-ray absorption spectroscopy, using an X-ray free electron laser in combination with an ultraviolet femtosecond laser, present desirable probes of coupled electronic and nuclear dynamics. In this work, we investigate the charge transfer dynamics of a donor-acceptor pair, which is widely used as a building block in low bandgap block copolymers for organic photovoltaics. We simulate the dynamics of the benzothiadiazole-thiophene molecule upon photoionization with a vacuum ultraviolet (VUV) pulse and study the potential of probing the subsequent charge dynamics using time-resolved X-ray absorption spectroscopy. The photoinduced dynamics are calculated using on-the-fly nonadiabatic molecular dynamics simulations based on Tully's Fewest Switches Surface Hopping approach. We calculate the X-ray absorption spectrum as a function of time after ionization at the Hartree-Fock level. The changes in the time-resolved X-ray absorption spectrum at the sulfur *K*-edge reveal the ultrafast charge carrier dynamics in the molecule occurring on a femtosecond time scale. These theoretical findings anticipate that ultrafast time-resolved X-ray absorption spectroscopy using an X-ray probe in combination with a VUV pump offers a new approach to investigate the detailed dynamics of organic photovoltaic materials.

## I. INTRODUCTION

The charge transfer from a donor to an acceptor plays a central role in photoinduced processes in both natural and artificial light harvesting systems. Therefore, achieving fundamental insight into the charge transfer dynamics is essential for many applications. One of the challenges associated with that comes from the atomic nature of these dynamics, which occurs on very short length and time scales, angstroms to nanometers and femtosecond to picoseconds. The detailed exploration of photoinduced processes requires experimental methods that are sensitive to both the electronic and nuclear degrees of freedom. Time-resolved, pump-probe techniques involve a nonstationary state initiated by a pump pulse and probed by means of a suitable probe pulse arriving with some time delay. However, pump-probe spectroscopy with visible light does not always yield a clear interpretation of the underlying dynamics. X-ray free-electron lasers, capable of producing femtosecond pulses of X-rays, are promising tools for enabling investigations of few-femtosecond nonequilibrium dynamics.^1–3^ The use of X-rays is appealing, because of not only their atom specificity due to localization of core transitions but also their ability to probe ultrafast dynamics.^4^ There are a growing number of time-resolved experiments for probing ultrafast nonadiabatic dynamics in photoexcited molecules.^5–13^ Of these, time-resolved X-ray absorption spectroscopy (TRXAS) has the advantage of detecting the local geometric structure of the system under study and, at the same time, the underlying electronic structure changes that drive the structural dynamics.^14–18^

Only a few theoretical studies have investigated the opportunities of TRXAS for studying ultrafast dynamics in molecules.^18–22^ For instance, a theoretical study of the use of ultrafast pre-edge TRXAS for probing nonadiabatic effects in the molecular dynamics of photoexcited molecules was done by Neville and co-workers.^20^ They investigated the sensitivity of the calculated TRXAS spectra to both geometric distortions and the electronic character of the initial state, demonstrating the potential of TRXAS for probing excited-state nonadiabatic molecular dynamics. Here, we use the localization of core orbitals, resulting in atomic-site specificity of TRXAS to track ultrafast charge transfer dynamics in real space. We study the time evolution of the location of the valence hole by exploiting the well-separated core-level absorption edges of the two sulfur atoms in benzothiadiazole-thiophene (BT-1T) and the atomic site-specificity of X-ray spectroscopy.

**FIG. 1.**
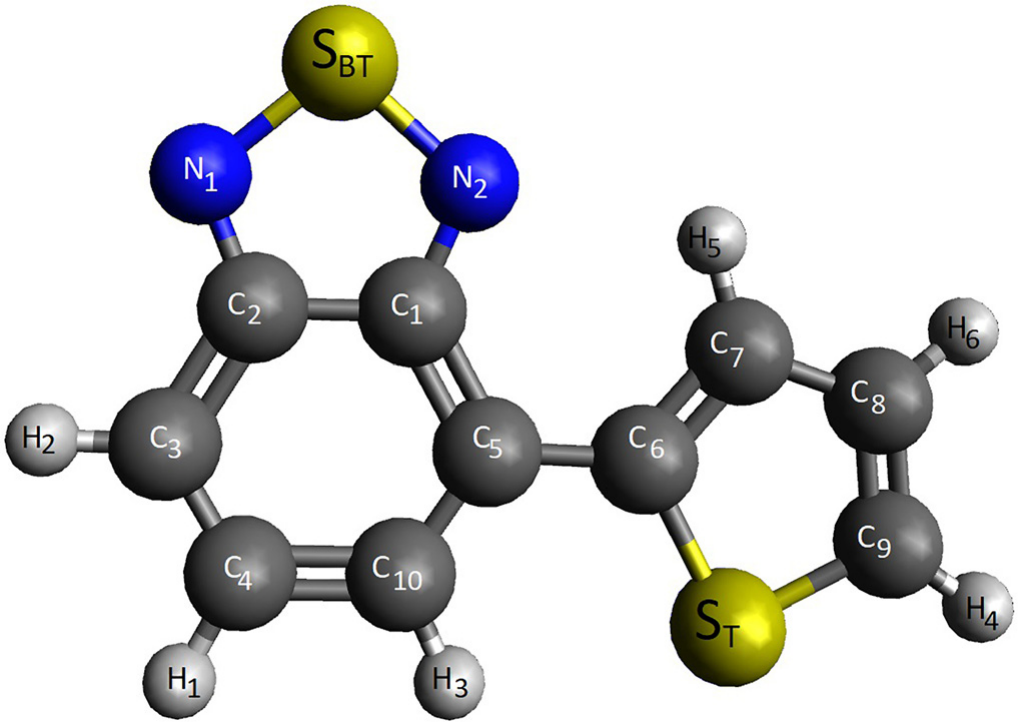
Schematic of the BT-1T molecule highlighting the two sulfur atoms.

BT-nT (n being the number of thiophene units) is one of the favored combinations of monomers in the light absorbing polymers^23–26^ (Fig. 1). Easy synthesis, flexible processing, and a bandgap matching the solar spectrum makes *π*-conjugated polymers attractive as structural building blocks in organic photovoltaic (OPV) applications.^27–30^ A typical polymer solar cell, as one of the most attractive types of OPV, has an active layer composed of a *π*-conjugated polymer as an electron-donating component and another polymer or a small molecule as an electron-accepting component.^31,32^ To achieve a desired bandgap, the polymer is often composed of two or more appropriate monomers with different electron affinities. Both the BT and T units in BT-1T contain a sulfur atom (shown as S_BT_ and S_T_ in Fig. 1). The different chemical environments of these sulfur atoms give rise to distinct core-level binding energies and, consequently, distinguishable X-ray absorption signals. This prototypical conjugated monomer therefore exemplifies a wide class of *π*-conjugated polymers, with two sulfur atoms in distinctly different chemical environments.

The functioning of a polymer solar cell depends on the successful dissociation of sunlight-generated electron-hole pairs into charge carriers and further requires migration of these pairs in the electron-donating polymer toward a donor-acceptor interface.^33,34^ We here present a strategy to study hole carrier dynamics of donor-acceptor type conjugated materials containing sulfur using the model monomer compound BT-1T in the gas phase. In a previous work, Scarongella *et al.*^35^ showed that studying the fragments of the polymer chains can give rise to fundamental insight into the charge transfer character of oligomeric/polymeric systems. By using time-resolved femtosecond transient absorption spectroscopy, they found that charge transfer relaxation in their model polymer compound occurs on time scales of a few picoseconds. In order to study the ultrafast dynamics in detail, we here consider a TRXAS scenario employing a vacuum ultraviolet (VUV) pump pulse beam for initial photoionization in combination with a tender X-ray probe pulse with an energy range near the sulfur *K*-edge (∼2.5 keV with tuning capability). A VUV photon will be absorbed by one of the electrons in the valence orbitals and thereby generates an ionized state by leaving a valence hole behind. Absorption of the X-ray light promotes an electron from the *K*-shell into the previously created valence hole and is thereby sensitive to the distance of the valence hole to the atom in which the *K*-shell is localized. Instead of the photoexcitation process relevant to photovoltaic applications, we here investigate the simpler process of photoionization as a model for the isolated hole dynamics in the case of photoexcitation.

After ionization through absorption of a single VUV photon, the molecule is vibrationally excited and the molecular geometry starts to rearrange. The electronic subsystem dynamics follow the nuclear rearrangement adiabatically, as long as electronic states are energetically separated. When electronic states come close to each other, nonadiabatic effects become relevant, which promotes the transfer of electronic excitation energy to the vibrational degrees of freedom. This process describes an electronic rearrangement that shifts the initially created charge in the molecule from one spatial position to another. We demonstrate that an X-ray absorption spectrum recorded at different time delays shows changes of spectral features that can be attributed to the ultrafast charge dynamics in the molecule which occur on a time scale of tens of femtoseconds.

## II. METHODS

### A. Nonadiabatic molecular dynamics

In order to obtain a time-resolved X-ray absorption spectrum of the VUV-pumped BT-1T molecule, we conduct mixed quantum-classical molecular dynamics simulations employing Tully's Fewest Switches Surface Hopping (FSSH) approach. FSSH is based on classical propagation of the nuclei and transitions between electronic quantum states.^36–38^ Starting from the equilibrium geometry of BT-1T in the ground electronic state, quasiclassical sampling is employed to generate 100 initial conditions (atomic coordinates and momenta). Each of the 100 initial conditions is then independently propagated with a time step of 0.5 fs and a total propagation time of 400 fs. Switching between the electronic potential energy surfaces is determined via probabilities calculated on-the-fly according to the FSSH algorithm. If a stochastic hop is accepted, the momenta of nuclei are scaled along the nonadiabatic coupling vector to conserve the total energy. We do not employ any decoherence corrections as these are not relevant for the irreversible electronic decay processes addressed in this work. The velocity Verlet algorithm^39^ is employed for propagating the nuclei. The implementation follows previous work by Subramanian *et al.*^40^

### B. Electronic structure

All the electronic-structure and X-ray-absorption calculations are carried out using the XMOLECULE toolkit,^41^ at the level of the restricted closed-shell Hartree-Fock (RHF) method and using the 6-31G Gaussian basis set. In order to have a consistent and, at the same time, efficient way to model all valence-hole states relevant for this study, we employ Koopmans' theorem, i.e., the potential energy surface of the ionized state (*N*−1 electrons) with a vacancy in orbital *i* is given by $E_{i}=E_{\text{RHF}}-\varepsilon_{i}$, where *ε_i_* is the energy of the *i*th occupied orbital and *E*_RHF_ is the ground-state RHF energy of the neutral (*N*-electron) system. We note that as long as the RHF method remains valid for the neutral ground state and we address holes in the outer valence, this model should give a qualitatively and semiquantitatively correct picture of the electronic structure and the dynamics in the hole state. The accuracy of the applied electronic structure method is further discussed in the supplementary material. This approach has been tested before in several previous studies addressing ionized state dynamics.^42–44^ We therefore expect this method to provide a model that is sufficient for the qualitative understanding of the molecular dynamics and its impact on the X-ray absorption spectrum. The energy gradients and nonadiabatic coupling matrix elements for the ionized states are calculated analytically based on the coupled perturbed Hartree-Fock (CPHF) theory,^45–47^ which is part of a new implementation in the XMOLECULE toolkit. The molecule is in the gas phase, and no solvent environment is considered in the current study.

The electronic-structure calculation shows that the four highest occupied molecular orbitals (MOs) in the BT-1T molecule are relatively close in energy at the equilibrium geometry of BT-1T in the ground electronic state. The considered valence orbitals and their corresponding energies are illustrated in Table I. The visualization of the molecular orbitals indicates that the four highest occupied orbitals have different degrees of contribution on the two sulfur atoms. This observation is quantified by the partial population of the respective orbitals on the two sulfur atoms, given in the last two rows of Table I. The partial hole populations (*P_h_*) on sulfur atoms S_BT_ and S_T_ are calculated based on the linear combination of atomic orbitals,

$$P_{h}^{S_{\text{BT},T}}=\sum_{\mu }^{{\text{onS}}_{\text{BT},T}}\sum_{\nu }C_{\mu h}S_{\mu \nu }C_{\nu h},$$ (1)

where *μ* and *ν* are the atomic basis function indices and $C_{\mu h}$ and $S_{\mu \nu }$ are the molecular orbital coefficients and overlap matrix elements, respectively. The sum over *μ* runs only over the basis functions on either S_BT_ or S_*T*_ (note that because of the selection rules, X-ray absorption involves excitation only to *p*-type orbitals). Whereas the HOMO orbital is not located on either of the two sulfur atoms, the HOMO-1 orbital has a significant proportion located on the sulfur atom S_*T*_. The orbitals HOMO-2 and HOMO-3 have little contribution from basis functions on sulfur atom S_*T*_ and some population on sulfur atom S_BT_.

**TABLE I.** Orbital energies (*E_b_*), isosurfaces, and partial hole populations on the sulfur atoms S_BT_ and S_*T*_ for the least bound orbitals of the BT-1T molecule at the ground state equilibrium geometry.

MO	HOMO	HOMO-1	HOMO-2	HOMO-3
*E_b_* (eV)	8.10	9.60	10.06	10.31
Isosurface (±0.01 au)	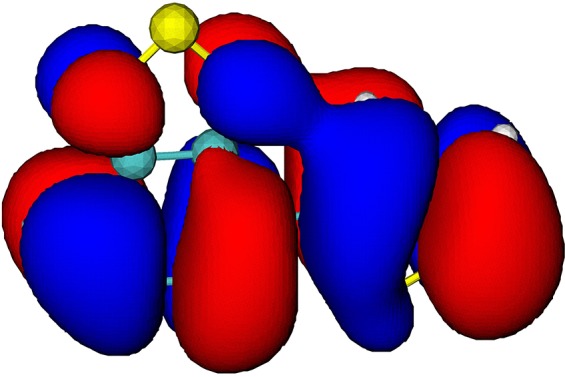	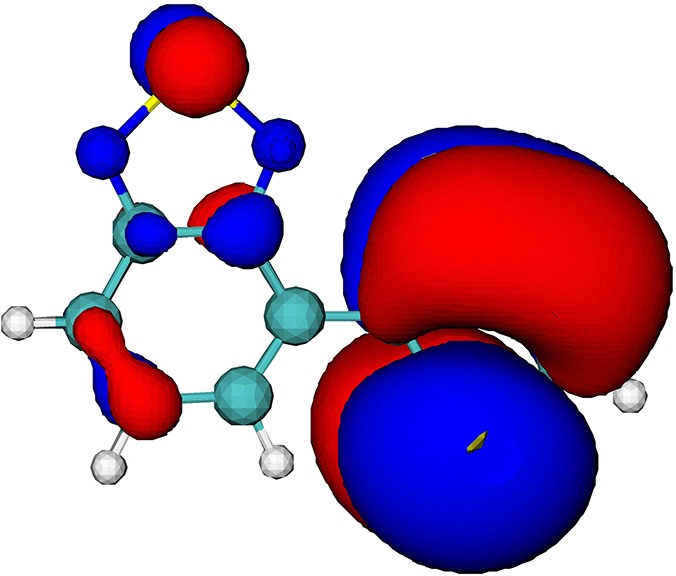	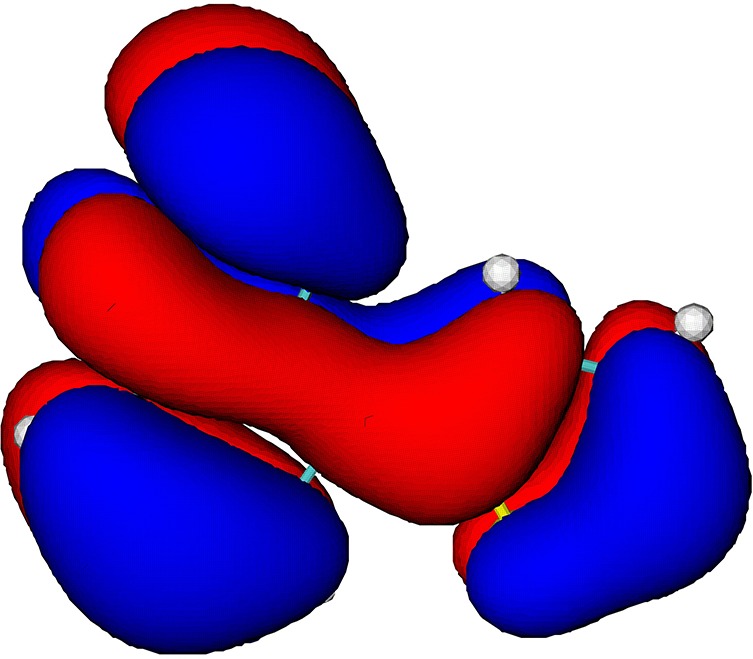	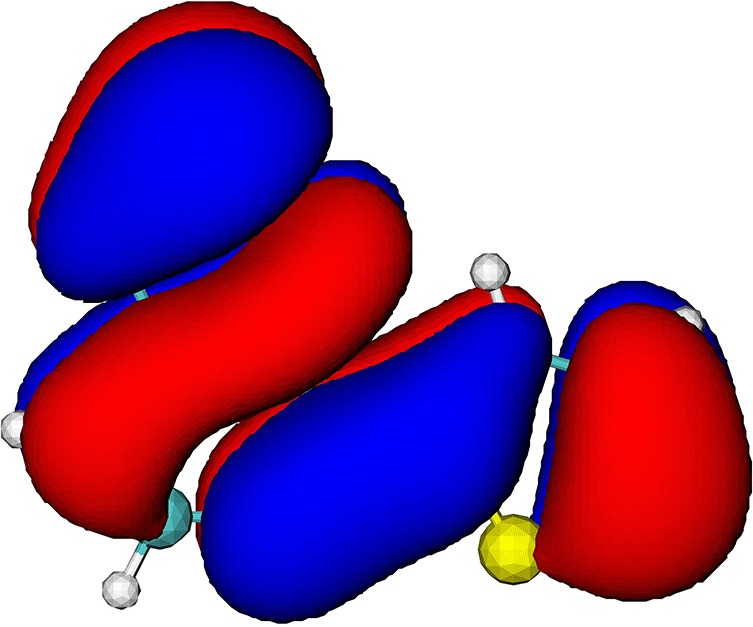
Part. Pop. on S_BT_	0.000	0.006	0.248	0.256
Part. Pop. on S_*T*_	0.006	0.611	0.014	0.000

### C. Photoabsorption cross sections

The cross section for bound-to-bound transitions (X-ray absorption), $\sigma_{a}$, from molecular orbital (MO) *ϕ_i_* to *ϕ_f_* (with energy eigenvalues *ε_i_* and *ε_f_*, respectively), is given in length form and employing the independent-electron picture by^48^

$$\sigma_{a}=\frac{4\pi^{2}}{3}\alpha \omega_{\text{in}}\sum_{\mu =x,y,z}|\langle \phi_{f}|\hat{\mu }|\phi_{i}\rangle {|}^{2}\delta (\varepsilon_{f}-\varepsilon_{i}-\omega_{\text{in}}),$$ (2)

where the sum runs over the three dipole directions μ=x,y,z and *α*, $\omega_{\text{in}}$, and $\left\langle \phi_{f}|\hat{\mu }|\phi_{i}\right\rangle$ are the fine-structure constant, the energy of the incoming photon, and the transition dipole matrix element, respectively. In Eq. (2), we have averaged over molecular orientations with respect to the polarization axis of the X-ray light.

## III. RESULTS AND DISCUSSION

**FIG. 2.**
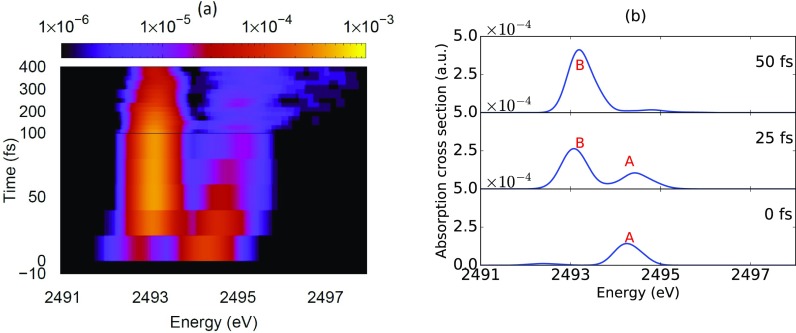
X-ray absorption spectra of a BT-1T molecule after photoionization of the HOMO-3 orbital. (a) Time-resolved X-ray absorption spectra (on a false color log-scale) as a function of time. Note that the time scale is not linear to stress variation with smaller time delays before 100 fs. (b) X-ray absorption spectrum at the selected delay times.

To study the dynamics induced by valence ionization, different ionized states are created by the removal of an electron from each of the four highest valence orbitals. Figure 2(a) shows the time evolution of the sulfur *K*-edge X-ray absorption spectrum following VUV photoionization of the HOMO-3 orbital. The spectra have been obtained by convolution with a Gaussian function with a standard deviation of *σ* = 0.2 eV to account for finite lifetime effects. We inspect in our calculation the specific energy window below the excitation threshold for the neutral molecule, because in the neutral molecule, X-ray absorption resonances associated with sulfur *K*-shell excitation are located within just a few electronvolts below the S 1s ionization threshold (2503.14 and 2505.00 eV for the two sulfur atoms within the current framework). The resonances that we are considering are at least 8 eV below the S 1s ionization threshold of the neutral molecule. Therefore, the neutral molecule does not display any resonant features in the spectral region that we are considering. For this reason, we note that at a negative time delay t < 0 (before the pump), there is no resonant X-ray absorption signal in this energy window (black part at negative time delays). This energy range is very advantageous for the experimental data collection since the X-ray absorption in a cation is distinguishable from X-ray absorption in a neutral system, as it has been demonstrated in several studies before.^49–51^ At t ≥ 0, two distinct features can be seen in the time evolution of the absorption spectrum in Fig. 2(a). The first feature is a peak around 2494.2 eV (labeled A) that appears with the arrival of the pump pulse. After a short delay (<20 fs), a second feature appears in the region around 2493.1 eV (labeled B) and peak A starts to become weaker. Beyond the 50 fs time delay, the intensity of peak B goes down but with a much slower rate than peak A. At the position of peak A, we can see some remaining low absorption for t ≥ 100 fs. In Fig. 2(b), snapshots of the spectrum at selected times are shown. Peak A and peak B can be directly attributed to the X-ray absorption on sulfur atoms S_BT_ and S_*T*_, respectively. Because of the different chemical environment, S_BT_ has a slightly higher *K*-edge binding energy, which gives rise to absorption at higher X-ray energies. S_*T*_ has a lower *K*-shell binding energy, and therefore, the X-ray absorption is at slightly lower energies.

**FIG. 3.**
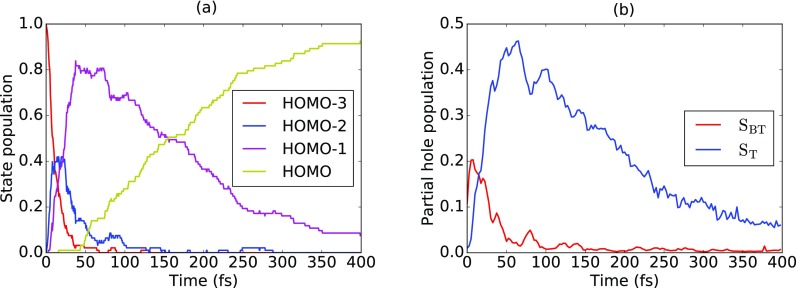
(a) Relaxation dynamics and (b) time evolution of partial hole population [Eq. (1)] on the two sulfur atoms after ionization of the HOMO-3 orbital.

The quick changes in the X-ray absorption spectrum can be understood from the electronic state populations from the FFSH calculations. Figure 3(a) shows the time evolution of the electronic state population after initial ionization of the HOMO-3 orbital. A very fast decay of the population in the HOMO-3 hole state with a half-lifetime of 8 fs can be seen. Subsequently, the HOMO-2 hole state and later the HOMO-1 hole state are populated. The decrease in the population of the HOMO-1 hole state after 50 fs leads to an increase in the cationic ground state (HOMO) population. This picture is also confirmed by inspecting the time evolution of the population of the valence hole on the two sulfur atoms as shown in Fig. 3(b). Initially, the hole is located close to S_BT_. Within 50 fs, it is transferred to S_*T*_ and later leaves the vicinity of both sulfur atoms. The changes in the absorption spectrum therefore directly reflect the movement of the valence hole from the vicinity of sulfur atom S_BT_ to sulfur atom S_*T*_ within less than 20 fs. The subsequent decrease in absorption peak B can be directly linked to the population of the HOMO hole state, in which the vacancy is not in the vicinity of either of the two sulfur atoms. From the orbital populations given in Table I, it can be inferred that the short-lived HOMO-3 and HOMO-2 hole states lead to X-ray absorption on sulfur atom S_BT_, whereas the later populated state (hole in HOMO-1) gives rise to absorption on S_*T*_. For a more detailed analysis of the X-ray absorption spectrum following VUV photoionization of the HOMO-3 orbital, see the supplementary material, Figs. S4 and S5.

**FIG. 4.**
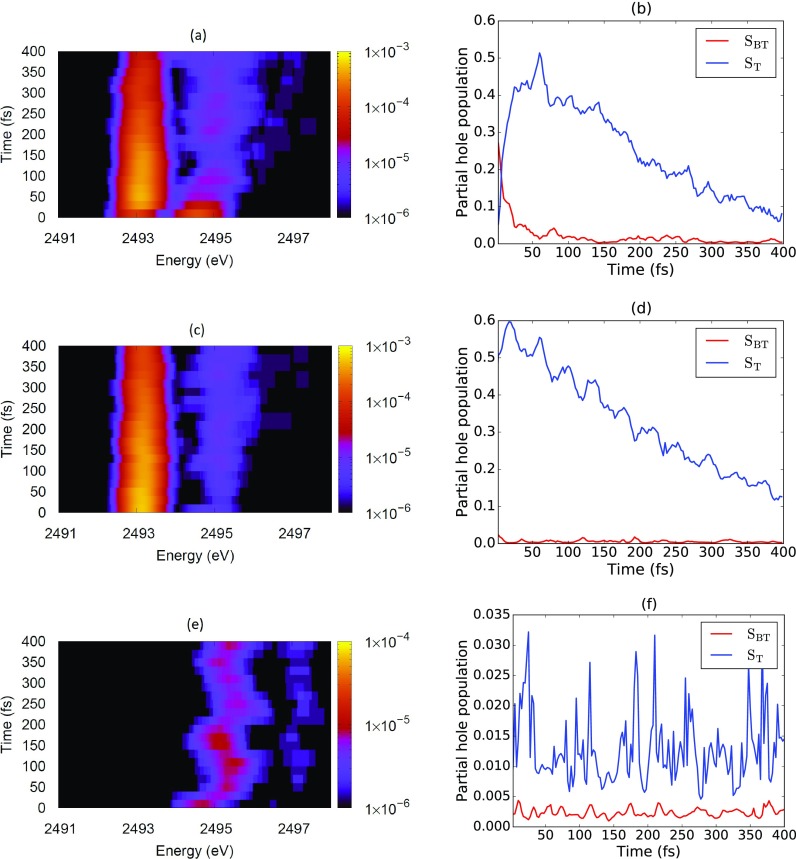
(a), (c), and (e) show the time-resolved X-ray absorption spectra and (b), (d), and (f) show the time evolution of the partial hole population after photoionization of (top) HOMO-2, (middle) HOMO-1, and (bottom) HOMO orbitals.

Figure 4 shows that the relation between hole population dynamics and the X-ray absorption signal holds in a similar manner also for initial ionization on the HOMO-2, HOMO-1, and HOMO orbitals. We specifically inspect the X-ray absorption spectrum after ionization on the HOMO orbital. The calculated X-ray absorption spectrum and the partial hole population on the two sulfur atoms after photoionization of the HOMO orbital are shown in Figs. 4(e) and 4(f), respectively. The spectrum in Fig. 4(e) shows a slight oscillation in the absorption signal with almost constant absorption strength. In this case, the hole is in the HOMO orbital, and thus, the absorption $S_{T}\to$ HOMO occurs at a higher energy relative to $S_{T}\to$ HOMO-1 [Fig. 4(c)]. As expected from the low overlap of the HOMO orbital with the two sulfur atoms (see Table I), the absorption is very low (note that the scale in the color bar is smaller by a factor 10 relative to Fig. 2). An even weaker signal can be seen at higher absorption energies, which can be identified as excitation from S_BT_ to the HOMO hole. The time-dependent hole populations in Fig. 4(f) show the changes of the hole populations on S_BT_ and S_*T*_, indicating that the valence hole undergoes certain fluctuations even in the electronic ground state of the cation. Variations in the absorption signal over time shown in Fig. 4(e) are the result of the dynamical evolution of the vibrationally hot molecule. Moreover, the dynamics following photoionization of the HOMO orbital can be compared with the dynamics following photoionization of the HOMO-3 orbital after long delay times, when the electronic state has relaxed to the ground state (hole in the HOMO orbital). The key difference, however, is that in the latter case, the molecule has much more vibrational energy. Compared to the late-time X-ray absorption signal after ionization of the HOMO-3 orbital, the spectrum after ionization of the HOMO orbital is much sharper. This observation suggests that the amount of vibrational energy in the molecule can be inferred from the particular changes in the X-ray absorption (near-edge) spectrum.

**FIG. 5.**
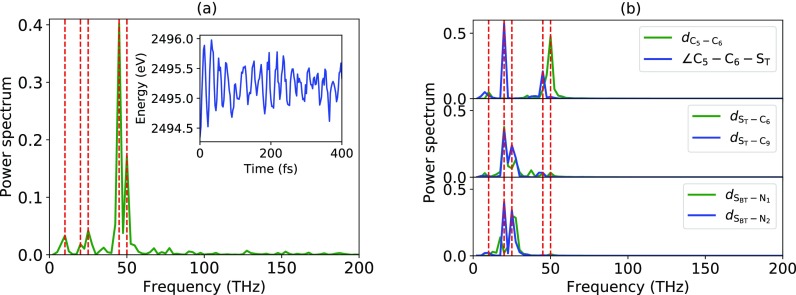
Fourier transform power spectrum of (a) the peak position of the X-ray absorption signal in Figs. 4(b) and 4(e) and the oscillation of the selected structural parameters. The red lines in (a) and (b) highlight the same set of frequencies.

We have conducted further analysis of the oscillations in the absorption signal for the simulations starting with a hole in the HOMO orbital. To that end, we have analyzed the Fourier transform, $f(\nu )=\int_{0}^{T}\;\text{exp}\;(i2\pi \nu t)f(t)$, of the peak position of the X-ray absorption signal *f*(*t*). The power spectrum, ${\left|f(\nu )\right|}^{2}/\sum_{\nu }{\left|f(\nu )\right|}^{2}$, of the peak position in the X-ray absorption spectrum is shown in Fig. 5(a). In the figure, dominant frequencies are highlighted with dashed lines. In order to link these oscillations with vibrations of the molecular geometry, we also conducted a Fourier analysis of the selected structural parameters. For our analysis, we consider specific coordinates that describe the local chemical environment around the two sulfur atoms as well as the bond connecting the two units in BT-1T (see Fig. 1). The frequencies that describe oscillations of the bond distance $d_{C_{5}{-C}_{6}}$ and the angle $∠C_{5}{-C}_{6}{-S}_{T}$ are shown in Fourier analysis in the top panel of Fig. 5(b). The power spectra for oscillations of the bonds $d_{S_{T}{-C}_{6}}$ and $d_{S_{T}{-C}_{9}}$ in thiophene and the bonds $d_{S_{\text{BT}}{-N}_{1}}$ and $d_{S_{\text{BT}}{-N}_{2}}$ in benzothiadiazole are shown in the middle and bottom panels of Fig. 5(b), respectively. As can be seen, the X-ray absorption spectrum oscillates with the same frequencies as the investigated bond parameters. We therefore conclude that the observed oscillations in the X-ray absorption signal can be attributed to specific vibrations in the molecule. This finding demonstrates that TRXAS also allows us to resolve local geometrical changes in the molecule.

## IV. CONCLUSION

The lack of a fundamental understanding of charge transfer dynamics in organic photovoltaics has long been recognized as a barrier for the further development of these complex devices. Therefore, we have undertaken a study to establish the theoretical foundation for experiments that will allow us to fully elucidate the mechanisms and dynamics of charge carrier transfer in organic heterojunctions. We demonstrate here that time-resolved X-ray absorption using an X-ray probe in combination with a VUV pump allows us to address the ultrafast carrier dynamics in molecular building blocks used for such devices with sensitivity toward both electronic and nuclear structures. As a model building block for donor-acceptor type polymers, we study BT-1T. After ionization in the HOMO-3 valence orbital, the electronic state quickly relaxes to the ground state (hole in the HOMO) and the molecule ends up in a highly excited vibrational state. During this relaxation process, the valence hole, initially created by VUV ionization, is transferred from one end (S_BT_) of the molecule to the other (S_*T*_). We demonstrate that this charge transfer can be inspected by X-ray absorption spectroscopy exploiting its atomic site-specificity. This technique can, therefore, provide new insight that will help to optimize the design of organic photovoltaic devices. We note that in a realistic experiment, the pump step requires a more detailed consideration. The characteristic signal associated with a particular hole can be identified either via inspection of the X-ray absorption spectrum with varying pump laser wavelengths or via coincident detection of the photoelectron. The work to implement the experiments proposed in this manuscript and to extend our methodology to model polymeric systems where absorption takes place in the visible light spectrum is in progress.

## SUPPLEMENTARY MATERIAL

See the supplementary material for additional figures and tables to support the electronic structure and X-ray absorption spectroscopy results, as mentioned in the text.
